# Effect of Iron Limitation, Elevated Temperature, and Florfenicol on the Proteome and Vesiculation of the Fish Pathogen *Aeromonas salmonicida*

**DOI:** 10.3390/microorganisms10091735

**Published:** 2022-08-27

**Authors:** Tobias Kroniger, Mina Mehanny, Rabea Schlüter, Anke Trautwein-Schult, Bernd Köllner, Dörte Becher

**Affiliations:** 1 Institute of Microbiology, Department of Microbial Proteomics, Center for Functional Genomics of Microbes, University of Greifswald, 17489 Greifswald, Germany; 2 Helmholtz Institute for Pharmaceutical Research Saarland, 66123 Saarbrücken, Germany; 3 Department of Pharmacy, Saarland University, 66123 Saarbrücken, Germany; 4 Department of Pharmaceutics and Industrial Pharmacy, Faculty of Pharmacy, Ain Shams University, Cairo 11566, Egypt; 5 Imaging Center of the Department of Biology, University of Greifswald, 17489 Greifswald, Germany; 6 Institute of Immunology, Friedrich-Loeffler-Institute, Federal Research Institute for Animal Health, 17493 Greifswald-Insel Riems, Germany

**Keywords:** *Aeromonas salmonicida*, iron limitation, temperature, antibiotic, florfenicol, outer membrane vesicles, proteomics, subcellular fractionation

## Abstract

We analyzed the proteomic response of the Gram-negative fish pathogen *A. salmonicida* to iron limitation, an elevated incubation temperature, and the antibiotic florfenicol. Proteins from different subcellular fractions (cytosol, inner membrane, outer membrane, extracellular and outer membrane vesicles) were enriched and analyzed. We identified several iron-regulated proteins that were not reported in the literature for *A. salmonicida* before. We could also show that hemolysin, an oxidative-stress-resistance chaperone, a putative hemin receptor, an M36 peptidase, and an uncharacterized protein were significantly higher in abundance not only under iron limitation but also with an elevated incubation temperature. This may indicate that these proteins involved in the infection process of *A. salmonicida* are induced by both factors. The analysis of the outer membrane vesicles (OMVs) with and without applied stresses revealed significant differences in the proteomes. OMVs were smaller and contained more cytoplasmic proteins after antibiotic treatment. After cultivation with low iron availability, several iron-regulated proteins were found in the OMVs, indicating that *A. salmonicida* OMVs potentially have a function in iron acquisition, as reported for other bacteria. The presence of iron-regulated transporters further indicates that OMVs obtained from ‘stressed’ bacteria might be suitable vaccine candidates that induce a protective anti-virulence immune response.

## 1. Introduction

The Gram-negative bacterium *Aeromonas salmonicida* is one of the major fish pathogens and the causative agent of furunculosis disease, resulting in high mortality and economic losses within the salmonid aquaculture industry. Many virulence factors of *A. salmonicida* have been reported to contribute to its pathogenicity. This includes the S-layer (or A-layer), which consists of the virulence array protein A (VapA) that is involved in the adhesion to macrophages [1], iron and heme acquisition systems [2,3,4,5], hemolysin/aerolysin [6], nucleases [6], lipases [6], proteases [6,7], chitinases [8], and other proteins involved in adhesion [9,10]. Further, *A. salmonicida* possesses a functional type-three secretion system (T3SS) [11,12,13] with several effector proteins [14,15,16]. Structural components of the T3SS are mainly encoded on the large pAsa5 plasmid [17,18]. The T3SS can be lost by insertion-dependent rearrangements on the pAsa5 plasmid [19], which can be triggered by stressful growth conditions such as high cultivation temperatures [17,19,20].

In the beginning, measures to handle furunculosis in the aquaculture industry were based on antibiotics treatment, e.g., florfenicol [21], but due to observed resistance phenomena, efforts were made to develop vaccines. The vaccines that are currently used generally consist of inactivated *A. salmonicida* [22,23] or subunit vaccine approaches applied via intraperitoneal or intramuscular injection [24,25]. However, these vaccinations are labor-intensive, require a certain size of fish, and may sometimes induce unwanted severe side effects [22,26].

Recently, vaccines have been tested using bacterial (outer) membrane vesicles (OMVs) [27,28]. These 20–400 nm-sized bubble-shaped entities can enclose proteins, DNA, RNA, peptidoglycan, lipopolysaccharides, and toxins [28,29,30,31,32]. It has been shown that the proteome of OMVs is dynamic and can change with environmental conditions and external stresses [33,34,35,36]. Bacterial OMVs are reportedly involved in various biological processes, including nutrient acquisition, intracellular communication, defense, stress response, biofilm formation, and virulence [27,37,38,39]. In terms of vaccine development, OMVs are an interesting platform as they present their antigens in their native conformation on the surface, cannot replicate, are highly immunogenic, are comparatively easy to bioengineer, and have already been successfully applied against other diseases such as bacterial meningitis [27,40,41,42]. It is known that *A. salmonicida* [43] and other bacteria in the genus *Aeromonas* [39,43] produce OMVs. While the OMV proteome has been investigated for other members in this genus [44], the protein composition of OMVs derived from *A. salmonicida* has not been studied yet.

Here, we utilize a bacterial subcellular fractionation approach, where proteins of the cytosol (Cyt), inner membrane (IM), outer membrane (OM), extracellular space (Extra), and outer membrane vesicles (OMVs) are enriched and analyzed. As the most abundant bacterial proteins are in the cytoplasm, this method of subcellular fractionation allows the reduction of sample complexity and, therefore, the dynamic range of the proteins and allows the identification of proteins with lower abundance [45,46]. Further, the determination of the experimental subcellular localization of proteins allows a deeper understanding of protein function and is crucial for potential future vaccine development efforts as membrane (lipo)proteins are especially highly immunogenic [47,48,49,50,51].

In this study, we analyze the effect of three stressors that the bacterium may face in the environment or the aquaculture industry on the proteome of *A. salmonicida* on a subcellular level. With this approach, the resulting data of the extracellular, membrane, and OMV proteomes will be of value for future rational vaccine designs as proteins in these localizations are at the host–pathogen interface and are potentially suitable vaccine candidates.

## 2. Materials and Methods

### 2.1. Bacterial Strain, Cultivation, Stress Conditions, and Harvest

The *Aeromonas salmonicida* subsp. *salmonicida* strain JF2267 (first described in [52]) was grown in quadruplicates in RPMI 1640 medium (Thermo Fisher Scientific, Waltham, MA, USA) supplemented with 50 µM FeCl_3_ and 100 µM citric acid in a 13 °C temperature water bath under shaking at 160 rpm (‘Ctrl’). Samples referred to as ‘FeLim’ were not supplemented with FeCl_3_ and citric acid. Samples referred to as ‘AB’ were supplemented with 0.5 µg/mL florfenicol (MedChemTronica, Stockholm, Sweden) during log-phase (OD_600_—0.3–0.4). Samples referred to as ‘Temp’ were grown at 19 °C. Growth curves for the stress conditions are visualized in Figure S1. For the analysis, bacteria were harvested at an OD_600_ of 0.9–1 by centrifugation at 10,000× *g* for 20 min at 4 °C. The bacterial pellet was used to isolate the cytosolic, inner membrane, and outer membrane protein subcellular fractions. To remove the remaining bacteria, the supernatant was filtered twice using 0.45 µm PES bottle-top filter membranes (VWR) and used to prepare the extracellular fraction. For the OMV isolation, due to a higher yield of OMVs, bacteria were harvested by centrifugation at 10,000× *g* for 20 min at 4 °C after reaching the stationary phase for at least 3 h. The OMV-containing supernatant was filtered using a 0.45 µm bottle-top filter membrane to remove the remaining bacteria and processed as described in ‘Preparation of OMV protein fraction’.

### 2.2. Preparation of Cytosolic, Inner Membrane, and Outer Membrane Protein Fractions

Bacterial cell pellets were resuspended in TE buffer (10 mM Tris-HCl, 1 mM EDTA, pH 7.25), and bacteria were disrupted via sonication in 5 cycles at −0.55 W in 0.7 s intervals for 1 min on ice. The total energy input was 0.7 kJ. Afterward, the cell extract was centrifuged for 10 min at 15,000× *g* and 4 °C to remove cell debris, and the supernatant was transferred into a new reaction tube and ultracentrifuged at 100,000× *g* for 1 h at 4 °C to pellet bacterial membranes. The supernatant contained the cytosolic proteins referred to as ‘Cyt’. The pellet was washed with HEPES (10 mM, pH 7.4) to remove potential contaminants and ultracentrifuged again. Afterward, the pellet was resuspended in 1 % (*w*/*v*) n-Lauroylsarcosine in 10 mM HEPES, which selectively solubilizes outer membrane proteins, as described by [53], and incubated for 30 min at 37 °C under shaking and ultracentrifuged for 1 h at 100,000× *g* and 4 °C subsequently. The supernatant contained the proteins of the inner membrane, referred to as ‘IM’. The pellet was washed in 10 mM HEPES (pH 7.4) and ultracentrifuged for 1 h at 100,000× *g* at 4 °C. The pellet contained the proteins of the outer membrane, referred to as ‘OM’. Prepared fractions were stored at −20 °C.

### 2.3. Preparation of OMV Protein Fraction

The filtered supernatant was concentrated ~20-fold using tangential flow filtration (Äkta flux, GE Healthcare, Chicago, IL, USA) with a nominal molecular weight cut-off of 100 kDa (UFP-100-C-3X2MA, Cytiva, Marlborough, MA, USA) to volumes that can be used in ultracentrifugation. Samples were ultracentrifuged afterward at 100,000× *g* for 3 h at 4 °C. The pellet was washed in PBS to remove potential contaminants and ultracentrifuged again for 3 h at 100,000× *g* and 4 °C. The outer membrane vesicle containing the pellet was resuspended in PBS; the fraction is referred to as ‘OMV’ and was stored at −20 °C.

### 2.4. OMV Nanoparticle-Tracking Analysis

The particle size distribution and yield of bacterial outer membrane vesicles were measured using nanoparticle-tracking analysis (NTA, LM-10, Malvern, UK), as described earlier [33]. Briefly, samples were diluted up to 1:1000 in filtered PBS to keep the particle concentration within the recommended range for reproducibility of the NanoSight LM-10 microscope. Around 200 µL of diluted OMVs were introduced into a green laser-illuminated chamber. Each sample was measured twice, in which three high-sensitivity 30 s videos at a camera level of 13–15 were recorded, then processed with NanoSight 3.1 software. Technical replicates were averaged.

### 2.5. S-Trap Protein Digestion and Peptide Fractionation

The S-Trap protein digest was performed according to the manufacturer’s protocol (ProtiFi) with minor modifications. Protein concentrations were determined by BCA assay according to the manufacturer’s instructions (Thermo Fisher Scientific). For the protein digest, 20 µg of protein of the Cyt, IM, OM, and OMV fractions was mixed 1:1 with 2× lysis buffer (10% SDS, 100 mM TEAB, pH 7.55). Afterward, proteins were reduced in 20 mM DTT for 10 min at 95 °C and alkylated in 40 mM IAA for 30 min in the dark. Samples were acidified by the addition of phosphoric acid to a final concentration of 1.2% and diluted 1:7 with S-Trap binding buffer (90% methanol, 100 mM TEAB, pH 7.1). The proteins were digested with 1:50 trypsin in 50 mM TEAB for 3 h at 47 °C in S-Trap microcolumns and the peptides were eluted from the columns using 50 mM TEAB, followed by 0.1% aqueous acetic acid and 60% acetonitrile containing 0.1% acetic acid. The peptides were dried using a vacuum centrifuge.

To reduce the sample complexity of the samples, basic reverse-phase peptide fractionation was performed as described previously [29]. In short, peptides were loaded onto in-house packed C18 micro spin columns (Dr. Maisch HPLC GmbH ReproSil pur C18, pore size 300 Å, particle size 5.0 µm) and eluted in eight fractions with increasing acetonitrile concentrations ranging from 5% to 50% in a high-pH solution (0.1% triethylamine). The eluates of fractions 1 and 5, 2 and 6, 3 and 7, and 4 and 8 were pooled. Peptides were dried using a vacuum centrifuge, resuspended in 20 µL buffer A (0.1% acetic acid), and stored at −20 °C until LC–MS/MS measurement.

### 2.6. Preparation of the Extracellular Protein Fraction and In-Gel Digestion

Extracellular proteins were enriched using StrataClean affinity beads (Agilent), as described before [54]. In brief, 20 µL of primed StrataClean beads were incubated with 10 mL of sterile-filtered bacterial culture supernatant in an overhead shaker overnight at 4 °C. The next day, the bead suspension with bound proteins was centrifuged for 45 min at 10,000× *g* and 4 °C. Afterward, the pellet was dried using a vacuum centrifuge, and the proteins were separated from the beads by SDS-PAGE. The separation was performed with 130 V until the solvent front traveled for roughly 3 cm. The gel was fixated and Coomassie-stained, and the lanes were cut in three pieces of equal size and tryptically digested. The dried peptides were resuspended in 10 µL Aq. dest. and desalinated using C18 ZipTips according to the manufacturer’s protocol (Merck Millipore). Afterward, peptides were resuspended in 20 µL buffer A (0.1% acetic acid) and stored at −20 °C until LC–MS/MS measurement.

### 2.7. Bioinformatic Tools

#### 2.7.1. PSORTb

PSORTb is a bioinformatic web-based online tool that predicts protein localizations based on their amino acid sequence [55]. As input for the prediction, the UniProt proteome of the *A. salmonicida* subsp. *salmonicida* strain M22710-11 (ID UP000232113, 4182 entries, download 25th June 2021) was used as the stored UniProt proteome of the used *A. salmonicida* subsp. *salmonicida* strain JF2267 (ID UP000186585) was marked to be redundant to the proteome of the M22710-11 strain at UniProt. The prediction was performed with standard settings: Organism: Bacteria; Gram-stain: Gram-negative. The resulting PSORTb prediction (version 3.0.2) differentiates between ‘unknown’, ‘cytoplasmic’, ‘cytoplasmic membrane’, ‘periplasmic’, ‘outer membrane’, and ‘extracellular’ protein localization and is available in Table S1.

#### 2.7.2. SignalP

SignalP is a bioinformatic web-based online tool that predicts the presence of signal peptides in proteins based on their amino acid sequence [56]. As input for the prediction, the UniProt proteome of the *A. salmonicida* subsp. *salmonicida* strain M22710-11 was used. The prediction was performed using the standard settings: Organism group: Gram-negative. The resulting SignalP prediction (version 5.0) differentiates between: ‘OTHER’: no predicted signal peptide; ‘SP-Sec/SPI’: standard secretory signal peptides transported by the Sec translocon and cleaved by signal peptidase I; ‘LIPO-Sec/SPII’: lipoprotein signal peptides transported by the Sec translocon and cleaved by signal peptidase II; ‘TAT-Tat/SPI’: Tat signal peptides transported by the Tat translocon and cleaved by signal peptidase. The predictions for the *A. salmonicida* proteome are available in Table S1.

#### 2.7.3. eggNOG

eggNOG is a bioinformatic web-based online tool that performs orthology-based functional annotation of proteins based on the amino acid sequence of proteins [57]. As input for the prediction, the UniProt proteome of the *A. salmonicida* subsp. *salmonicida* strain M22710-11 was used. The prediction was performed using the standard settings. The resulting eggNOG prediction (version 5.0) of the proteome is available in Table S1.

### 2.8. Mass Spectrometry Data Acquisition and Analysis

Tryptic peptides of the subcellular fractions were separated on an Easy nLC 1200 liquid chromatography system (Thermo Fisher Scientific) with a reverse-phase C18 column (in-house packed, inner diameter 100 µm, outer diameter 360 µm, length 200 mm, packed with Dr. Maisch ReproSil pur C18, pore size 120 Å, particle size 3.0 µm) and a column oven set to 45 °C. Peptides were loaded with 22 µL of buffer A (0.1% acetic acid) at 400 bar and subsequently eluted with a non-linear 100 min gradient (OM, Extra, and OMV fractions) or a non-linear 180 min gradient (Cyt and IM fraction) from 1% to 99% buffer B (95% acetonitrile with 0.1% acetic acid) at a constant flow rate of 300 nL/min. Eluting peptides were measured in an Orbitrap Elite mass spectrometer (Thermo Fisher Scientific) in a data-dependent mode. The MS1 scan was recorded in the orbitrap with a mass window of 300–1700 m/z and a resolution of 60,000. The 20 most intense precursor ions (ions with an unassigned charge or a charge of 1 were excluded) were selected for CID fragmentation with a collision energy of 35%. The resulting MS/MS spectra were measured by the linear ion trap.

The resulting raw-files were searched with MaxQuant software (version 2.0.1.0) [58] against the UniProt proteome of the *A. salmonicida* subsp. *salmonicida* strain M22710-11 (ID UP000232113, 4182 entries, download 25 June 2021). For detection of contaminations, the cRAP contaminants list was used. The search was performed with a maximum of two missed cleavages, oxidation (M) and acetylation (protein N-term) as variable modifications, and carbamidomethylation (C) as a fixed modification. Proteins were identified with a minimum of two peptides per protein group, with at least one unique peptide. Match between runs was enabled between biological replicates. For protein quantification, unique and razor peptides were used, and the label-free quantification (LFQ) calculation was performed separately for each of the enriched subcellular fractions.

The resulting data were analyzed with Perseus software (version 1.6.15.0) [59]. Data were filtered based on hits against the reverse database, identified by site and the contamination list of MaxQuant. For statistical testing, only proteins with quantitative data in at least 3 out of 4 replicates of a condition in one subcellular fraction were considered. To also consider proteins that are on/off regulated, missing values were imputed from the normal distribution, and two-sided Student’s t-tests with a false discovery rate of 0.05 were performed.

### 2.9. Transmission Electron Microscopy

Cells were fixed (1% glutaraldehyde, 4% paraformaldehyde, 0.2% picric acid, 50 mM sodium azide in 20 mM HEPES buffer) for 30 min at room temperature and then stored at 4 °C until further processing. Subsequent to embedding in low gelling agarose, cells were washed in washing buffer (20 mM cacodylate buffer pH 7, 10 mM calcium chloride) two times for 10 min each time, postfixed in 2% osmium tetroxide in washing buffer for 1 h, washed with deionized water for 5 min, washed with 0.05% sodium chloride two times for 5 min, and then stained with 2% uranyl acetate in 0.05% sodium chloride for 30 min. Cells were washed with deionized water three times for 5 min each time, and after dehydration in a graded series of ethanol (20%, 30%, 50%, 70%, 90% for 10 min each, 96% two times for 10 min, 100% ethanol three times for 10 min), the material was embedded in epoxy resin (formerly EPON 812). Sections were cut on an ultramicrotome (Reichert Ultracut, Leica UK Ltd., Milton Keynes, UK) and stained with 4% aqueous uranyl acetate for 3 min, followed by lead citrate for 30 s. After air drying, samples were analyzed with an LEO 906 transmission electron microscope (Zeiss Microscopy Deutschland GmbH, Oberkochen, Germany) at an acceleration voltage of 80 kV. For image acquisition, a Sharpeye wide-angle dual-speed CCD camera (Tröndle, Moorenweis, Germany) was used, operated by ImageSP software. Afterward, the micrographs were edited using Adobe Photoshop CS6.

### 2.10. Field Emission Scanning Electron Microscopy

The cells were filtered onto a 0.2 µm polycarbonate filter (0.2 µm, GTTP, Merck KGaA, Darmstadt), and cells adsorbed to this filter were fixed with a fixation solution (1% glutaraldehyde, 4% paraformaldehyde, 0.2% picric acid in 5 mM HEPES buffer) for 1 h at room temperature (RT) and then at 4 °C until further processing. Subsequently, samples were treated with 2% tannic acid in washing buffer (100 mM cacodylate buffer (pH 7.0), 1 mM calcium chloride) for 1 h, 1% osmium tetroxide in washing buffer for 1 h, and 1% thiocarbohydrazide for 30 min at RT, with washing steps in between. After treatment with 1% osmium tetroxide in washing buffer for 1 h at RT, the samples were washed three times in washing buffer for 5 min each and then dehydrated in a graded series of aqueous ethanol solutions (10%, 30%, 50%, 70% at 4 °C overnight, 90%) and in 100% ethanol on ice for 15 min each step. Before the final change of 100% ethanol, samples were allowed to reach room temperature and then critical-point-dried with liquid CO_2_. Finally, samples were mounted on aluminum stubs, sputtered with an approximately 10-nm-thick gold/palladium film, and examined with a Supra 40VP field emission scanning electron microscope (Carl Zeiss Microscopy Deutschland GmbH, Oberkochen, Germany) using an Everhart–Thornley SE detector and an in-lens detector at a 50:50 ratio at an acceleration voltage of 5 kV. All micrographs were edited using Adobe Photoshop CS6.

## 3. Results

### 3.1. Evaluation of the Subcellular Fractionation

**Figure 1:**
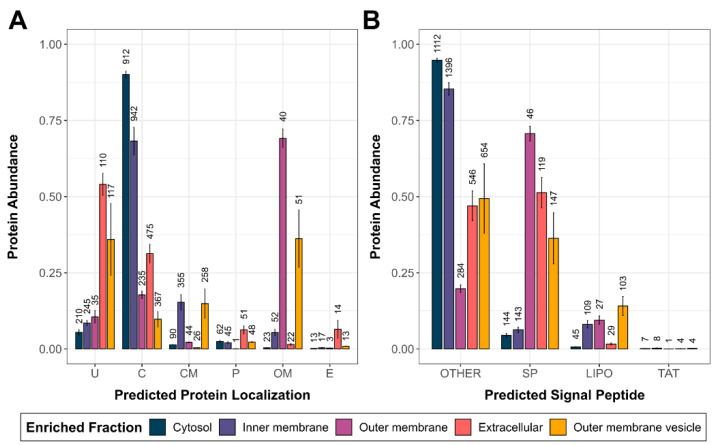
Overview of the predicted protein localizations and the presence of proteins with signal peptides within the enriched subcellular fractions of the Ctrl condition. (**A**) Quantified proteins were grouped according to their PSORTb-predicted protein localization (U—unknown; C—cytoplasmic; CM—cytoplasmic membrane; P—periplasmic; OM—outer membrane; E—extracellular). The protein abundances of these groups were normalized, and the distribution within the enriched subcellular fractions of the Ctrl condition was plotted. The number of quantified proteins within the enriched fractions is indicated above the bars. Error bars indicate standard deviations between replicates (*n* = 4). (**B**) Quantified proteins were grouped according to their SignalP-predicted presence of signal peptides (OTHER—no predicted signal peptide; SP—Sec translocon/signal peptidase I; LIPO—Sec translocon/signal peptidase II; TAT—Tat translocon/signal peptidase I). The protein abundances of these groups were normalized, and the distribution within the enriched subcellular fractions of the Ctrl condition was plotted. The number of quantified proteins within the enriched fractions is indicated above the bars. Error bars indicate standard deviations between replicates (*n* = 4).

First, the success and purity of the subcellular fractionation protocol were evaluated based on the protein abundances within the enriched subcellular fractions of the control (Ctrl) condition. For this evaluation, two bioinformatics tools were used. The tool PSORTb predicts the subcellular localization of a protein in silico based on the protein’s amino acid sequence [55]. The tool SignalP, on the other hand, can predict the presence of signal peptides of proteins in silico based on the amino acid sequence [56]. Protein quantities belonging to the same PSORTb-predicted protein localization (Figure 1A) or the same SignalP-predicted signal peptide (Figure 1B) were summed up and normalized by the total LFQ intensity in the respective enriched subcellular fraction.

While the cytosolic proteins were by far the most abundant in the Cyt fraction, the predicted cytosolic proteins were also highly abundant in the IM fraction, indicating an imperfect fractionation. Nevertheless, inner membrane proteins were enriched in abundance and number in the IM fraction compared to the other subcellular fractions. The OM fraction was highly enriched with outer membrane proteins, and the Extra fraction was comparatively enriched with extracellular proteins. In the Extra fraction, cytoplasmic proteins and proteins of unknown localization were also highly abundant. In the OMV fraction, proteins with unknown localization and predicted outer membrane proteins had the highest combined abundance, but inner membrane proteins were also comparatively abundant.

Next, similar to the PSORTb analysis, the presence and abundance of proteins with signal peptides in the subcellular fractions of the Ctrl conditions were examined (Figure 1B). While proteins in the Cyt and IM fractions were mainly without a signal peptide, proteins with a standard Sec/SPI secretion signal were enriched in the OM, Extra, and OMV fractions. Additionally, proteins with a lipoprotein secretion signal were comparatively more abundant in the IM, OM, and OMV fractions. Similar visualizations of the enriched proteins in the iron limitation (FeLim), elevated incubation temperature (Temp), and antibiotic (AB) conditions are available in Figures S2 and S3. Lists of the total protein identifications in the different conditions and the used bioinformatics tools (PSORTb, SignalP, eggNOG) are available in Table S1.

### 3.2. Influence of the Stress Conditions on the Composition of the Subproteomes of A. salmonicida

**Figure 2:**
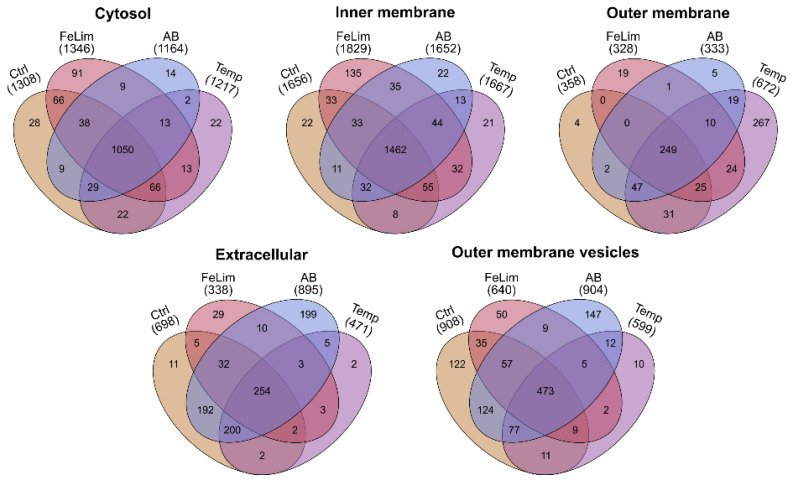
Overview of the overlap of quantified proteins between the stress conditions and the control in the enriched subcellular fractions. Shown are the number of proteins that were quantified in at least 3 out of 4 replicates in each condition in the enriched subcellular fractions and their respective overlap in quantified proteins with other conditions. Ctrl—control; FeLim—iron limitation; AB—antibiotic stress; Temp—elevated temperature stress.

Next, the proteomic response of the *Aeromonas salmonicida* strain JF2267 to the applied stress conditions and their impact on the subproteomes are described. Most of the quantified proteins were found in all of the applied conditions (Figure 2). Of all conditions, the highest total number of proteins were quantified in the Cyt and IM fractions. Nevertheless, differences in the number of quantified proteins between the stress conditions were observed. For example, the number of proteins in the OM fraction of the Temp condition was nearly doubled compared to the other conditions. Additionally, more proteins were quantified in the IM fraction of the FeLim condition compared to the other conditions. Similarly, more proteins were quantified in the Extra and OMV fractions of the Ctrl and AB conditions compared to the other conditions. Lists of the quantified proteins in each subcellular fraction of the stress conditions are available in Table S2.

**Figure 3:**
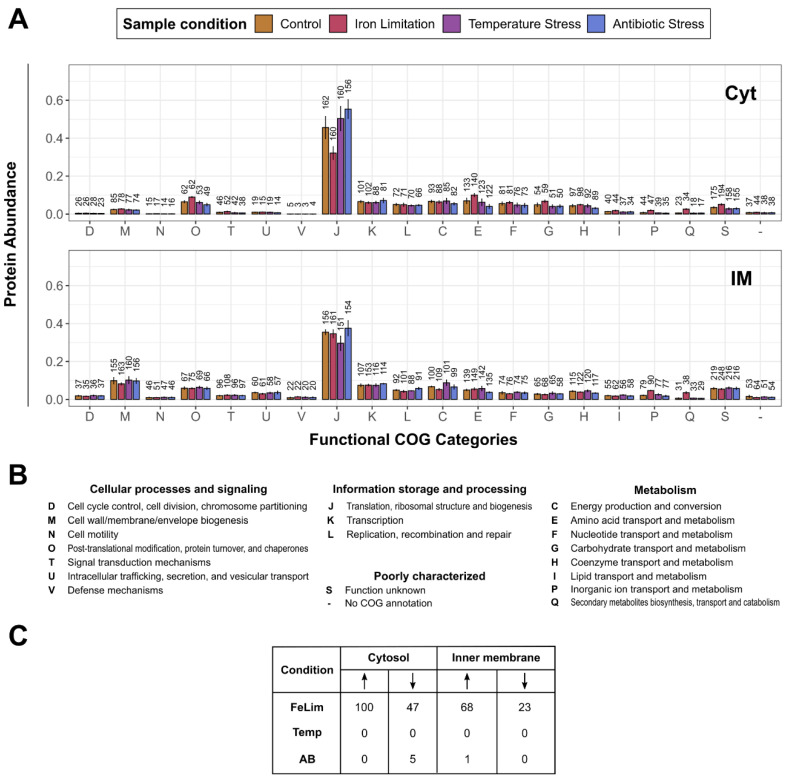
Overview of the functional protein compositions in the enriched cytoplasmic and inner membrane fractions. (**A**) Quantified proteins were grouped into functional categories that were predicted by eggNOG (description of the one-letter code in (**B**)). The protein abundances of each bin were normalized to the total protein abundances in the enriched fraction and condition. The distribution within the conditions in the enriched cytosol (Cyt) and inner membrane (IM) fractions were plotted. The number of quantified proteins within the conditions is indicated above the bars. Error bars indicate standard deviations between replicates (*n* = 4). (**B**) COG one-letter code descriptions. (**C**) The number of proteins with significantly changed abundance. Arrow pointing up: significantly upregulated; Arrow pointing down: significantly downregulated. Detailed data are available in Table S3.

**Figure 4:**
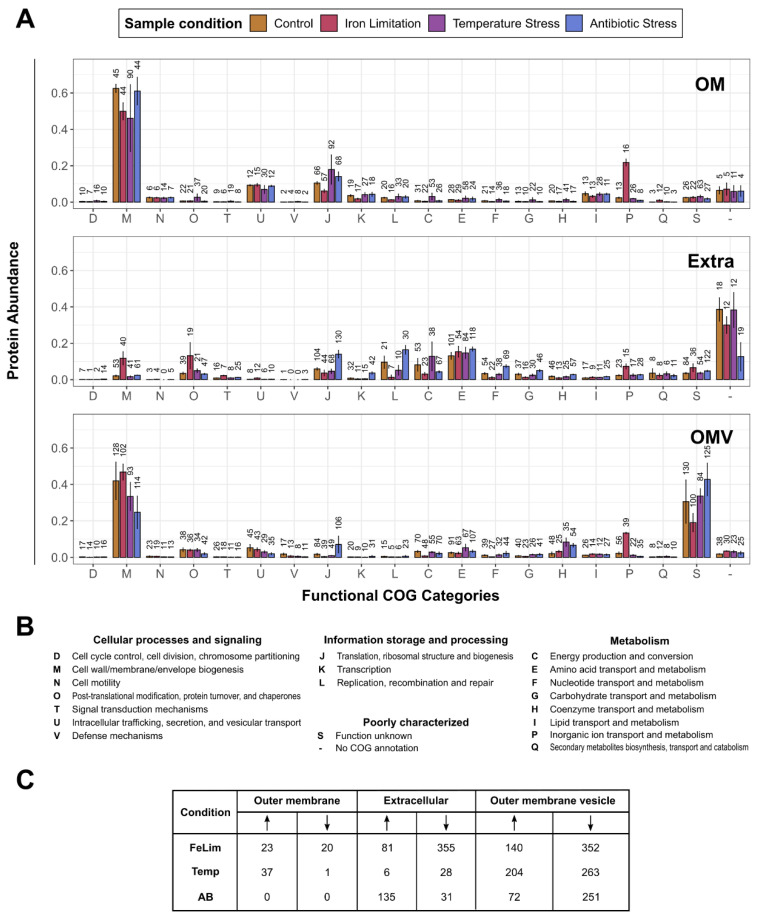
Overview of the functional protein compositions in the enriched outer membrane, extracellular, and outer membrane vesicle fractions. (**A**) Quantified proteins were grouped into functional categories that were predicted by eggNOG (description of the one-letter code in (**B**)). The protein abundances of each bin were normalized to the total protein abundances in the enriched fraction and condition. The distributions within the conditions in the enriched outer membrane (OM), extracellular (Extra), and outer membrane vesicle (OMV) fractions were plotted. The number of quantified proteins within the conditions is indicated above the bars. Error bars indicate standard deviations between replicates (*n* = 4). (**B**) COG one-letter code descriptions. (**C**) The number of proteins with significantly changed abundance. Arrow pointing up: significantly upregulated; Arrow pointing down: significantly downregulated. Detailed data are available in Table S3.

To pinpoint the function of proteins that had a changed abundance after the application of the stress conditions, functional clusters of orthologous group (COG) annotation were performed with eggNOG [57], and the differences in the quantitative protein distribution between the conditions were analyzed afterward. We further analyzed significant changes in protein abundances in response to the stress conditions for all enriched subcellular fractions. For this analysis, only proteins with quantitative data in at least three out of four replicates of a condition were considered. An overview of the number of proteins with significantly changed abundance for each fraction and condition is shown in Figure 3C and Figure 4C. Detailed tables of the significantly changed proteins, including protein names, q-values, and the imputed LFQ values used for the statistics, are provided in Table S3.

In the Cyt fraction, proteins involved in ‘J-Translation, ribosomal structure, and biogenesis’ were the most abundant proteins in all conditions (Figure 3A). In the FeLim condition, proteins of this functional category had a smaller share as many ribosomal proteins were significantly less abundant (Figure 3C, Table S3). Contrary, proteins involved in ‘O—Post-translational modification, protein turnover, and chaperones’, ‘E—Amino acid transport and metabolism’, ‘P—Inorganic ion transport and metabolism’, and ‘Q—Secondary metabolites biosynthesis, transport, and catabolism’ were found to be relatively more abundant in the Cyt and IM fractions of the FeLim condition than in the other conditions. Many of these proteins were significantly changed in abundance (Table S3). These proteins included several proteins involved in non-ribosomal peptide synthesis, iron transport and acquisition, hemolysin, aerolysin, and peptidases. Similar to the Cyt fraction, proteins categorized as ‘J—Translation, ribosomal structure, and biogenesis’ were highly abundant in all conditions of the IM fraction (Figure 3A). Proteins involved in ‘M—Cell wall/membrane/envelope biogenesis’ were increased in the number of identifications but were also in relative abundance in the IM fraction compared to the Cyt fraction. In the Temp and AB conditions, few or no proteins were significantly changed in abundance compared to the Ctrl condition (Figure 3C).

In the OM fraction, proteins involved in ‘M—Cell wall/membrane/envelope biogenesis’ had the biggest relative abundance in all conditions (Figure 4A), followed by ‘J—Translation, ribosomal structure, and biogenesis’ and ‘U—Intracellular trafficking, secretion, and vesicular transport’. While the number of identified proteins was comparable to the Ctrl condition, the relative abundance of proteins involved in ‘P—Inorganic ion transport and metabolism’ was increased drastically in the FeLim condition. Proteins with significantly increased abundance within this category (Figure 4C, Table S3) included several TonB-dependent (siderophore) receptors. In the Temp condition, high deviations in abundance and the number of identified proteins compared to other conditions were observed, and 37 proteins were found to be significantly increased (Figure 4C, Table S3), including hemolysin, peptidases, and TonB-dependent receptors. In the Extra fraction, proteins without a COG annotation were the most abundant group, even though only 12–19 proteins were quantified (Figure 4A). The most abundant protein in this fraction and this category was the S-layer protein (Q6EVH0). Proteins categorized as ‘E—Amino acid transport and metabolism’ were also abundant in the Extra fraction of all conditions. Major differences were observed in the FeLim and AB conditions compared to the control. In the FeLim condition, a relative increase in the abundance of proteins involved in ‘M—Cell wall/membrane/envelope biogenesis’, ‘O—Post-translational modification, protein turnover, and chaperones’, ‘P—Inorganic ion transport and metabolism’, and ‘S—Function unknown’ was observed. Significantly increased proteins in these categories included porins, non-ribosomal peptide synthetases, isochromatases, and proteases (Table S3). In contrast, proteins involved in all functions of ‘Information storage and processing’ were decreased in abundance in the FeLim condition and increased in the AB condition. Proteins involved in ‘F—Nucleotide transport and metabolism’ were also increased in relative abundance in the AB condition. In contrast, proteins involved in ‘C—Energy production and conversion’ and without COG annotation were decreased in abundance in the AB condition. In the OM fraction of the Temp condition, a type II secretion protein, hemolysin, a DJ-1 family chaperone, alpha-amylase, pantothenate synthetase, and an uncharacterized protein were significantly increased in abundance (Figure 4C, Table S3). In the OMV fraction, proteins involved in ‘M—Cell wall/membrane/envelope biogenesis’ and without COG annotation had the biggest share in abundance across all conditions (Figure 4A). In the FeLim condition, a major increase in abundance was observed for proteins involved in ‘P—Inorganic ion transport and metabolism’, while proteins categorized as ‘S—Function unknown’ were decreased in abundance. Proteins with significantly increased abundance in the FeLim OMVs included several TonB-dependent (iron) receptors, peptidases, porins, and a lot of proteins without known function (Figure 4C, Table S3). In the AB condition, proteins involved in ‘M—Cell wall/membrane/envelope biogenesis’ were found to be decreased in relative abundance, while proteins involved in ‘J—Translation, ribosomal structure, and biogenesis’ and ‘S—Function unknown’ were increased. Proteins involved in ‘H—Coenzyme transport and metabolism’ were found to be increased in abundance in both the AB and Temp conditions. These changes were also reflected in proteins with significantly changed abundances (Figure 4C, Table S3).

Overall, the condition with the highest number of proteins with significantly changed abundance was the FeLim condition. Most protein abundances that were found to be increased in the FeLim condition were non-ribosomal peptide synthetases, involved in siderophore biosynthesis/transport, other iron/heme transporters, TonB-dependent proteins, chitin-degrading proteins, superoxide dismutases, stress response proteins, proteases, peptidases, and many proteins with unknown function. Proteins with iron/heme or iron–sulfur clusters as cofactors were the main group of proteins that were found to be less abundant in the FeLim condition. In response to the antibiotic, significant changes in the proteome were mainly observed in the Extra and OMV fractions, where several cytoplasmic proteins, e.g., protein abundances involved in translation, ribosomal structure, and biogenesis, were increased. Interestingly, one ABC transporter (A0A6N4D0R0) was found to be significantly increased in abundance in the IM fraction and all replicates of the OM fraction during the AB condition but was not quantified in any replicate in any other condition in this study. In the Temp condition, no significant changes were detected in the Cyt and IM fractions. Proteins that were observed to be more abundant in the Temp condition were proteases, TonB-dependent proteins, transcriptional regulators, cell envelop proteins, a lysozyme inhibitor, chaperones, proteins with unknown function, and proteins involved in virulence (Table S3).

### 3.3. The Outer Membrane Vesicles of A. salmonicida

To determine whether the iron limitation, elevated incubation temperature, and the antibiotic conditions have an impact on the vesiculation of *A. salmonicida* besides the OMV proteome itself, nanoparticle-tracking analysis (NTA) was performed, which allows the determination of the concentration and size distribution of particles in the enriched fractions.

**Figure 5:**
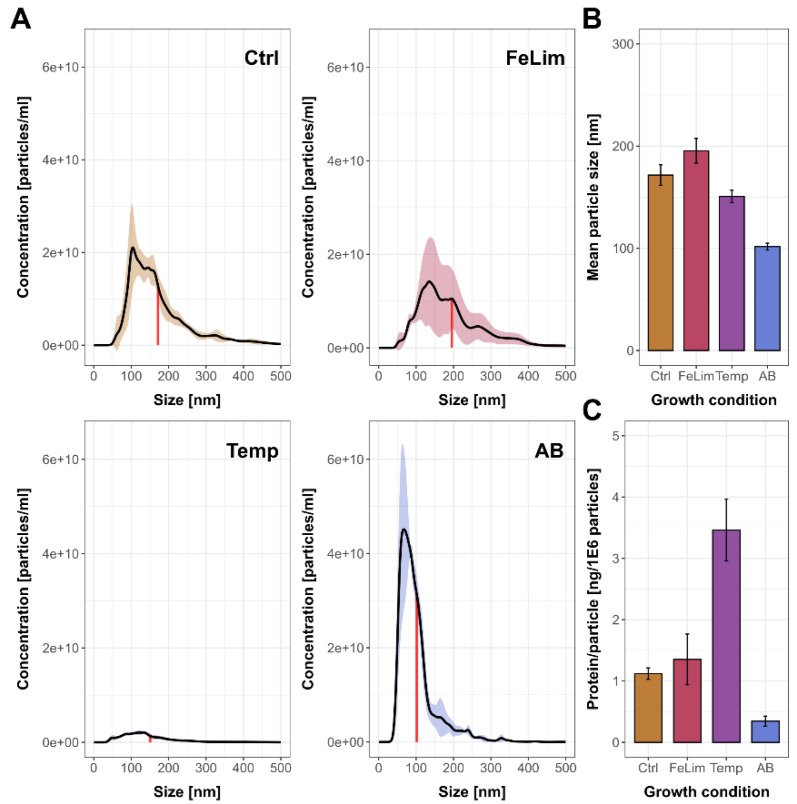
Nanoparticle-tracking analysis of *Aeromonas salmonicida* OMVs. (**A**) Size distribution and concentration determination of the outer membrane vesicle fraction by nanoparticle-tracking analysis (Ctrl—control; FeLim—iron limitation; Temp—elevated temperature; AB—antibiotic stress). The colored areas indicate the 75% confidence intervals of the data between replicates, and the red line indicates the mean particle size of the respective condition. (**B**) Mean particle sizes within the outer membrane vesicle fraction of the applied conditions. (**C**) Protein-to-particle ratio within the outer membrane vesicle fraction of the applied conditions. OMV protein concentration determinations were obtained by BCA assay before S-Trap protein digest for mass spectrometry analysis. Error bars indicate standard deviations between replicates.

**Figure 6:**
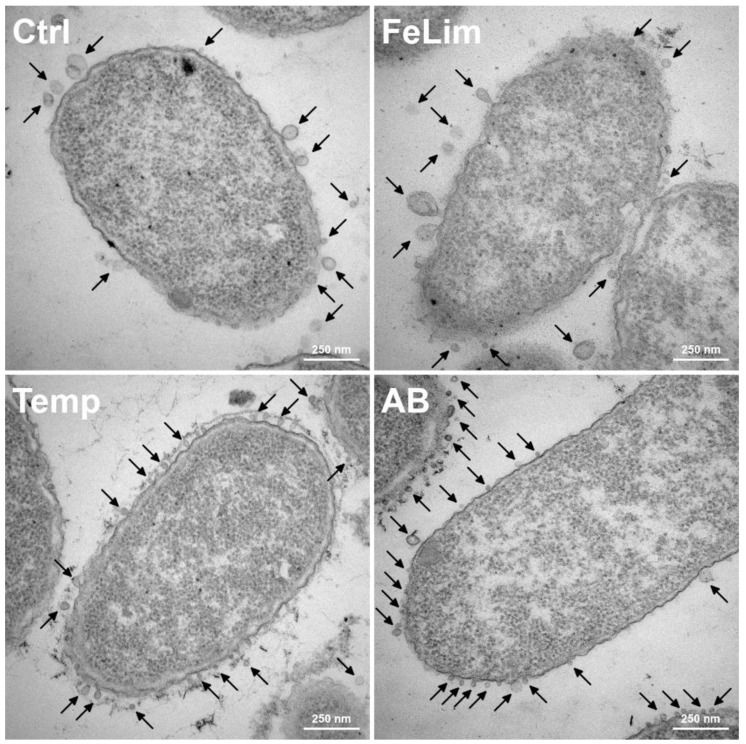
Transmission electron micrographs of *Aeromonas salmonicida* under control (Ctrl), iron limitation (FeLim), elevated temperature (Temp), and antibiotic stress (AB) conditions. Arrows indicate OMVs. Scale bars = 250 nm.

While particle concentrations of the OMVs were comparable under Ctrl (Figure 5A), FeLim, and AB conditions, fewer particles were observed in the Temp condition. Particles enriched during the FeLim condition were slightly bigger on average (Figure 5B) but also more diverse in their size distribution between replicates compared to the Ctrl condition (Figure 5A). The particle size of OMVs enriched in the AB condition was drastically decreased and more heterogenous compared to the other conditions. OMVs enriched during the Temp condition were slightly smaller but had a higher protein per particle ratio (Figure 5C). The differences in the size of the vesicles were visible in transmission electron micrographs (TEMs) of the bacterium (Figure 6). Bacteria in all conditions had intact cell membranes, and the blebbing of the OMVs was visible (indicated by arrows in Figure 6). While the shedding of OMVs, in general, was also observed in scanning electron micrographs (SEMs) (Figure S4), the size of the OMVs seemed to be more uniform in the TEMs compared to the SEMs. In the SEMs, it was visible that the bacterial surface was fully covered in OMVs. Further, nanotube-like structures that were connecting bacterial cells were observed in all conditions (Figure S4).

**Figure 7:**
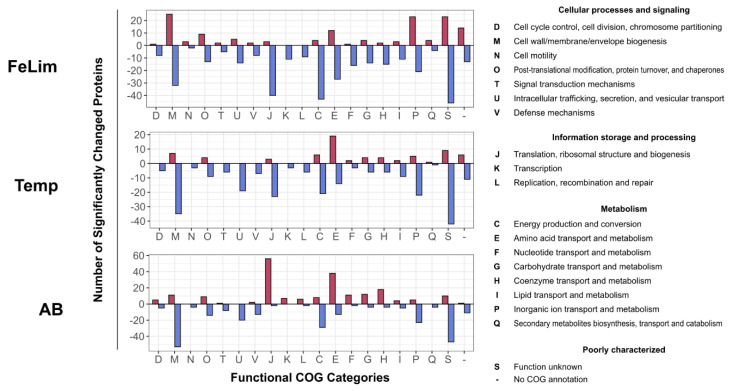
The number of significantly changed proteins for the OMVs derived in the stress conditions. Significantly changed proteins were grouped according to their eggNOG-predicted functional COG categories (FeLim—iron limitation; Temp—elevated temperature stress; AB—antibiotic stress). On the right side are the descriptions of the COG one-letter code.

As already mentioned, the OMV proteome differed drastically between the applied conditions (Figure 2 and Figure 4C). For the analysis of such a high number of significantly changed proteins, proteins were assigned to their functional COG category and the number of proteins significantly changed within this category was visualized (Figure 7).

In all of the applied conditions, many proteins assigned to ‘M—Cell wall/membrane/envelope biogenesis’, ‘T—Signal transduction mechanisms’, ‘U—Intracellular trafficking, secretion, and vesicular transport’, ‘V—Defense mechanisms’, ‘C—Energy production and conversion’, ‘P—Inorganic ion transport and metabolism’, and ‘S—Function unknown’ were significantly less abundant compared to Ctrl OMVs (Figure 7). In the FeLim condition, numerous proteins categorized as ‘M—Cell wall/membrane/envelope biogenesis’, ‘U—Intracellular trafficking, secretion, and vesicular transport’, ‘P—Inorganic ion transport and metabolism’, and ‘S—Function unknown’ or without COG annotation were found to be increased in abundance (Figure 7). In contrast, various proteins with functions in ‘J—Translation, ribosomal structure, and biogenesis’ and all other ‘Information storage and processing’ categories, ‘F—Nucleotide transport and metabolism’, ‘G—Carbohydrate transport and metabolism’, and ‘H—Coenzyme transport and metabolism’ were significantly less abundant. Many proteins assigned to ‘J—Translation, ribosomal structure, and biogenesis’ and other ‘Information storage and processing’ categories were significantly more abundant in the AB condition. Further, several proteins involved in metabolism, e.g., ‘E—Energy production and conversion’, ‘F—Nucleotide transport and metabolism’, ‘G—Carbohydrate transport and metabolism’, and ‘H—Coenzyme transport and metabolism’, were more abundant in the OMVs of the AB condition (Figure 7). In the Temp condition, mainly proteins involved in ‘E—Energy production and conversion’ were upregulated.

## 4. Discussion

In this study, the proteomic adaption of *A. salmonicida* to three external stressors that the bacterium may face in the environment or aquaculture was analyzed. For this, a subcellular fractionation approach was applied, which allows a deeper protein coverage and a more focused view on the significantly changed proteins within several localizations of the bacterial cell. In the Ctrl condition, *A. salmonicida* was cultivated in an iron-supplemented RPMI medium at 13 °C, which is the optimal growth temperature of Atlantic salmon, one of the hosts of *A. salmonicida*. The Ctrl condition served as a reference for the success and number of identified proteins for the subcellular fractionations and comparative proteomics with the stress conditions. In the first stress condition, bacteria were cultivated as in the Ctrl condition but without iron supplementation, resulting in an iron limitation stress that mimics an environment that the pathogen faces within the host. Iron acquisition is one of the key tasks of bacterial pathogens as the availability of iron in the host is scarce [60,61]. In the second stress condition, bacteria were cultivated at an elevated temperature (Temp) of 19 °C. This condition mimics a behavioral fever where fish change their thermal preference in response to an infection to amplify their innate immune response [62,63]. In the third stress condition, bacteria were cultivated as in the control condition, but 0.5 µg/mL of the antibiotic florfenicol was spiked in during the logarithmic growth phase. The florfenicol concentration of 0.5 µg/mL was chosen as this concentration has a measurable impact on the growth of *A. salmonicida* (Figure S5) while keeping the cells viable (Figure 6). Florfenicol is a primarily bacteriostatic broad-spectrum antibiotic that is licensed and used in aquaculture [64]. Additionally, all of these stressors have been reported to influence the OMV proteome and the shedding of OMVs in general [35,36,37,65,66]. The most important variations between the tested conditions are listed in Table 1.

### 4.1. Subcellular Fractionation

Overall, the applied protocol for subcellular protein enrichment was successful as proteins were quantitatively and qualitatively enriched in their predicted protein localizations compared to the quantitative data in the other enriched subcellular fractions (Figure 1A). This approach seemed to work best with cytosolic and outer membrane proteins, as the Cyt and OM fractions yielded the highest purity of proteins predicted to be in these localizations. The IM fractions yielded the highest numbers of identified proteins in all conditions (Figure 2). As the share of cytosolic proteins in this fraction is very high (Figure 1A), many of the cytosolic proteins seem to be co-enriched. However, when compared to the other enriched subcellular fractions, inner membrane proteins were enriched in the IM fraction. In the Extra fraction, proteins with a secretion peptide were the most abundant protein species. Nevertheless, proteins predicted to be cytosolic were also highly abundant in this fraction. This may be explained by a certain amount of cell lysis but also by proteins with a moonlighting function.

Bergh et al. analyzed putative moonlighting proteins that are present in *A. salmonicida* and other bacteria [67]. They listed 35 putative moonlighting proteins and hypothesized that these proteins may be secreted via OMVs. In fact, of the 35 putative moonlighting proteins, we identified 34 in the Extra fraction and 32 in the OMV fraction. However, several proteins were identified in the extracellular milieu of *A. salmonicida* that were not identified in the OMVs and vice versa. Additionally, the relative abundances of numerous proteins varied drastically between both subcellular fractions. In our data, 278 predicted cytosolic proteins were identified in both the extracellular space and the OMVs (Table S2). Of these, 197 were exclusively quantified in the extracellular fraction and 89 in the OMV fraction. While some cytoplasmic-predicted proteins such as the malate dehydrogenase (A0A6N4CWX9) or the 50S ribosomal protein L7/L12 (A0A6N4CQL8) were found highly abundant in the Extra fraction, they were not found in the OMVs. Vice versa, for example, the protein HflC (A0A6N4D0Q9), ATP-dependent Clp protease (A0A6N4CVB7), and Beta-1,4-galactosyltransferase (A0A6N4CXK7) were found highly abundant in the OMV fraction but not in the Extra fraction. The presence of cytoplasmic proteins within OMVs can, in general, be explained by so-called outer–inner membrane vesicles (OIMVs), which are double-bilayered MVs described for Gram-negative bacteria [68,69]. In our data, inner membrane proteins were highly abundant in the OMV fraction (Figure 1A), giving stronger evidence for the presence of OIMVs. OIMVs could also explain the differences in size and the higher abundance of cytosolic proteins observed in the OMV fraction of the AB condition (Figure S2), as other classes of antibiotics are reported to induce the bacterial SOS response, which may lead to cell lysis and the production of OIMVs [68]. Overall, we see a correlation between predicted cytoplasmic proteins in the extracellular and the OMV proteomes, as hypothesized by Bergh et al.; however, several examples show that these proteomes are quite different, and not all of these observations can be explained by the secretion of cytoplasmic proteins within OMVs. Our data demonstrate that the OMV proteome varies greatly between the conditions. This may show one of the biological roles of OMVs in bacteria, as they are reported to be involved in the fast modification of the bacterial cell surface proteome [70].

### 4.2. Response to the Iron Limitation

The acquisition of iron is essential for bacterial pathogens and often directly or indirectly coupled to virulence [5]. This is one of the reasons why several studies have covered the proteomic response of *A. salmonicida* to an environment with low iron availability in the past decades [2,3,4,71,72,73]. Ebanks et al. identified three proteins that were upregulated in response to low iron conditions [71]. These proteins were a 73 kD colicin receptor, a 76 kDa outer membrane heme receptor, and an 85 kDa ferric siderophore receptor. Our study confirms these results, as homologs of all three proteins (Q6RBX7; Q6RBX6; Q6SLH5) were found to be significantly upregulated. Najimi et al. described the siderophore biosynthesis cluster, which is essential for growth under iron limitation conditions [2]. We found all six proteins belonging to the cluster (A0A6N4CWC2; A0A6N4CYM2; A0A6N4CYP8; A0A6N4CW88; A0A6N4CZ10; A5I8G0) significantly increased in abundance in response to the FeLim condition. Similarly, Najimia et al. described the heme uptake genes of *A. salmonicida* [4]. Of the nine proteins described in this cluster, we found seven proteins (Q6RBX6; A5I8G4; A0A6N4CZL3; A5I8G7; A5I8G8; A5I8G9; A5I8G3) to be significantly increased in response to iron limitation. One protein (A5I8G5) was quantified but not significantly changed, and one protein (A0A6N4D019) was not identified. In another study, Najimi et al. performed a Fur titration assay and identified Fur-regulated genes [3]. Our study found 5 of the 13 Fur-regulated proteins to be significantly changed during iron limitation (A5I8G1; A0A6N4CYM8; Q6SLH5; A0A6N4CYM9; A0A6N4CNL5), 5 were quantified but not significantly regulated (A5I8H2; A0A6N4CS56; A0A6N4CUJ8; A5I8H6; A0A6N4CSB4), and 3 could not be identified (A0A6N4CSR5; A5I8H4; A5I8H3).

In our work, we were able to report several additional proteins with significantly increased abundance in a low iron environment (Table S3). Most of these proteins are involved in (TonB-dependent) iron/siderophore transport and the biogenesis of these proteins. Further, many proteins involved in cell wall synthesis, remodeling, and degradation were significantly increased. Other proteins with significantly increased abundance are involved in the virulence of the bacterium and may outline the quality of the presented dataset. For example, *A. salmonicida* has two coded superoxide dismutases (SODs) (SodA-Q7WYM8: SodB-Q7WYN0). While SodA was found to be significantly increased during iron limitation and low abundant or missing in other conditions, SodB was identified in the other conditions but low abundant or missing during iron limitation. The environmental-dependent SOD expression is due to different cofactored prosthetic metals as SodA is cofactored by manganese and SodB is cofactored by iron, as described previously [74]. Similarly, most of the proteins that were significantly decreased in abundance in the FeLim condition have iron, heme, or iron–sulfur clusters as cofactors. This also included, for example, cytochrome c proteins, which were generally less abundant except for two cytochrome c proteins that were induced during FeLim (A0A6N4CR45; A0A6N4CND1). The environmental availability of iron has also been reported in other bacteria to influence cytochrome c regulation [75]. Other proteins that may be involved in processes during infection and were significantly increased in abundance during FeLim were chitin-binding and degrading proteins (A0A6N4CXW3; A0A6N4CWG8; A0A6N4D0S7), hemolysin (A0A6N4CSV6), aerolysin (A0A6N4CNV5), flagellar/pili/fimbriae proteins (A0A6N4CWZ1, A0A6N4CZ16, A0A6N4CVX1, A0A6N4CS92), lipase /acyltransferase proteins (A0A6N4CSA9, A0A6N4CVH0), galactose binding proteins (A0A6N4CQ53), and a glycosidase (A0A6N4CMG9). Interestingly, 49 of the proteins with significantly increased abundance had no annotation or known function according to UniProt (Table S3). Some of these were only detected during FeLim (A0A6N4D0E4; A0A6N4CZU3; A0A6N4CTR0; A0A6N4CPH4; A0A6N4CU70), giving strong evidence that these proteins have functional or regulatory roles to cope with the FeLim condition and, therefore, potentially during infection.

The OMVs formed during the iron limitation had similar size and concentration as the OMVs derived during the control condition. Many iron-regulated (membrane) proteins involved in the iron acquisition, considered promising antigens, were only found during iron-limiting conditions on the OMVs. For example, of the 65 proteins Bergh et al. identified in the supernatant that have antigenic homologs in other bacteria and constitute candidates for a subunit vaccine [67], we identified 42 in the *A. salmonicida* OMVs during FeLim. Of the 24 outer membrane-associated proteins that may be suitable as a subunit vaccine candidate [67], we were able to identify 17 of them in the OMVs. Marana et al. formulated three subunit vaccine candidates consisting of 14 proteins in total [24]. All three subunit vaccines showed significantly lower mortalities compared to the control groups. The OMVs derived during our study in the FeLim condition contained seven of these proteins. Other conserved outer membrane proteins potentially suitable as vaccine candidates include the outer membrane protein assembly factor BamA, the TonB-dependent siderophore receptor, and the LPS assembly protein LptD [25]. All of them were identified in the OMV fraction during the FeLim condition. Therefore, OMVs of *A. salmonicida* derived under iron-limiting conditions may represent a suitable platform for the development of a new route of vaccination against furunculosis. Further, it has been shown that OMVs conduct biological functions in other bacteria, including the acquisition of iron [37,76]. The high number and quantity of iron transport proteins in *A. salmonicida* OMVs could indicate a similar biological function of *A. salmonicida* OMVs in the acquisition of iron.

### 4.3. Response to an Elevated Incubation Temperature

The speed and severity of the course of the furunculosis disease caused by *A. salmonicida* are associated with an increased water temperature [77]. While no proteins in the Cyt and IM fractions were found to be significantly changed in their abundance, several proteins were changed in the other subcellular fractions. This included proteins that play a role during the infection. In the extracellular space, amongst others, a pullulanase (A0A6N4CWY4), a hemolysin (A0A6N4CSV6), an oxidative-stress-resistance chaperone (A0A6N4D0J0), an alpha-amylase (A0A6N4CPK2), and an uncharacterized protein (A0A6N4CYU6) were significantly increased in abundance. An M36 peptidase (A0A6N4CZ72) was also more abundant but slightly below the significance threshold. In the outer membrane, a ligand-gated channel protein (putative hemin receptor) (A5I8G1) was found to be significantly increased in abundance. The hemolysin, the oxidative-stress-resistance chaperone, the ligand-gated channel protein (putative hemin receptor), the uncharacterized protein, and the M36 peptidase were significantly increased in the FeLim condition, indicating that these proteins can be regulated by both iron limitation and elevated temperature. That hemolysin can be regulated by temperature has already been shown for other members of the *Aeromonas* genus [78]. This result shows that the rapid disease progression experienced with a higher water temperature may be influenced by infection-relevant proteins as some of them are regulated by both elevated temperature and iron availability. This may be one way for the bacterium to bypass the behavioral fever that fish can induce when facing an infection [62,63].

While, for many bacteria, an increased temperature is associated with increased production of OMVs [38], we observed decreased OMV production with the elevated temperature, which has been reported for other cold-water-adapted bacteria [79]. The applied stress temperature of 19 °C is a temperature where the host experiences stress. *A. salmonicida* is not exclusively psychrophilic [80] but can also grow at higher temperatures. Therefore, 19 °C may not be high enough to lead to a higher vesiculation phenotype, as hypothesized. Whether this observation is caused by protein or lipid dynamics and whether higher vesiculation can be achieved with higher temperatures has to be clarified in more detailed comparative studies.

### 4.4. Response to Florfenicol

Florfenicol is a broad-spectrum antibiotic that is mainly used in veterinary medicine and aquaculture [64]. Even though florfenicol is considered a bacteriostatic, the data of the Extra fraction suggests that bacterial cell lysis is occurring as the number of cytosolic proteins and their abundances drastically increased compared to the control condition (Figure S2; Table S2). Most of these proteins were ribosomal proteins and were involved in translation, ribosomal structures, and biogenesis, which strengthens this hypothesis. Similar to the Extra fraction, proteins involved in these functions were also the proteins with the biggest increase in abundance in the OMV fraction. Together with the data of the NTA analysis, this might propose that the OMVs were derived through a different mechanism of the bacterial parent cell as their protein cargo not only differed greatly from the other conditions but the OMVs themselves were also drastically smaller (Figure 5). One explanation for this observation could be that the binding to the 50S ribosomal subunit and the resulting inhibition of protein synthesis by florfenicol result in a loss of regulation of membrane maintenance and leakage of cytosolic proteins and the formation of smaller OMVs with a different protein cargo. Similar to our results in other Gram-negative bacteria, chloramphenicol, which has an analogous mode of action to florfenicol [81], did not influence the amount of OMV production but significantly decreased OMV-associated Shiga toxin 2a and OMV cytotoxicity compared to other classes of antibiotics [66]. In another study, Devos et al. described the impact of antibiotics on the vesiculation of the Gram-negative bacterium *Stenotrophomonas maltophilia*. They found that after antibiotic treatment, a prophage was induced, which led to smaller and more heterogeneous MVs that enclosed more cytoplasmic proteins [68]. As some phage proteins are abundant proteins in the AB OMV fraction (Table S2), a similar effect may have been observed here for *A. salmonicida*. Our results emphasize the drastic effects of florfenicol on the OMV shedding and the OMV proteome. Both florfenicol [82] and OMVs [39] have been associated with an increased biofilm formation of *A. salmonicida*. Therefore, it would be interesting to see whether OMVs are involved in the observed effects of increased biofilm formation after florfenicol treatment of *A. salmonicida*.

## 5. Conclusions

In this study, we analyzed the proteomic adaption of *A. salmonicida* to three environmental stresses and were able to report quantitative data of roughly 2000 proteins in total per condition. The presented data provide new insights into the subcellular adaption of *A. salmonicida,* and we were able to expand the list of iron-regulated proteins of this important fish pathogen, which will be of value for future vaccine development efforts. Many of the iron-regulated proteins were found in the OMV fraction of the bacterium that was collected during iron-limiting conditions, indicating the potential of OMVs harvested under iron-limiting conditions for future vaccine research as many of the iron-regulated membrane proteins are considered promising antigens. After antibiotic treatment, the OMV size was drastically decreased and resulted in a more heterogenous vesicle population. We could also show that some proteins, including hemolysin, were not only significantly increased in an environment with iron-limiting conditions but also by an elevated incubation temperature. This highlights that some virulence factors of *A. salmonicida* are not only regulated by iron availability but also by an elevated water temperature that fish may prefer for the induction of behavioral fever.

## Figures and Tables

**Table 1:** Summary of the most important variations between the tested conditions.

	Iron Limitation	Temperature	Antibiotics
Excerpt of the proteomics results	Several upregulated IROMPs (TonB-dependent proteins, iron/siderophore transporters, hemolysin, aerolysin)	Upregulated hemolysin, putative hemin receptor, oxidative-stress chaperon, pullulanase, uncharacterized protein	Increased ribosomal proteins in extracellular space and outer membrane vesicles
Vesiculation (Mean particle size of control: 172 nm)	Slightly increased vesicle size (Mean particle size: 195 nm)	Slightly decreased vesicle size (Mean particle size: 151 nm)	Decreased vesicle size (Mean particle size: 102 nm) potentially due to prophage induction
Vesiculation amount	Comparable vesiculation amount to control condition	Less vesiculation compared to control	Comparable vesiculation amount to control condition

## Data Availability

The mass spectrometry proteomics data have been deposited with the ProteomeXchange Consortium via the PRIDE partner repository [83] with the dataset identifier PXD034641.

## Supplementary Materials

The following supporting information can be downloaded at: https://www.mdpi.com/article/10.3390/microorganisms10091735/s1. Figure S1: Growth curves of *Aeromonas salmonicida* grown under different stress conditions; Figure S2: Proteins quantified in the applied conditions were clustered according to their PSORTb predicted protein localization (U—unknown; C—cytoplasmic; CM—cytoplasmic membrane; P—periplasmic; OM—outer membrane; E—extracellular) for each of the enriched subcellular fractions. The protein abundancies of these clusters were normalized and the distribution within the enriched subcellular fractions was plotted. The number of quantified proteins in the experimental condition is indicated above the bars. Error bars indicate standard deviations between replicates (*n* = 4); Figure S3: Quantified proteins were clustered according to their SignalP predicted presence of signal pep-tides (OTHER—no predicted signal peptide; SP-Sec/SPI: ‘standard’ secretory signal peptides transported by the Sec translocon and cleaved by signal peptidase I; LIPO-Sec/SPII: lipoprotein signal peptides transported by the Sec translocon and cleaved by signal peptidase II; TAT-Tat/SPI: Tat signal peptides transported by the Tat translocon and cleaved by signal peptidase). The protein abundancies of these clusters were normalized and the distribution within the enriched subcellular fractions of the applied conditions were plotted. of the applied conditions within the enriched subcellular fractions (A—Cytosol; B—Inner membrane; C—Outer membrane; D—Extracellular; E—Outer membrane vesicle). The number of quantified proteins within the enriched fractions are indicated above the bars. Error bars indicate standard deviations between replicates; Figure S4: Scanning electron micrographs of *Aeromonas salmonicida* under control (Ctrl), iron limitation (FeLim), elevated temperature (Temp) and antibiotic stress (AB) conditions. Scale bars = 1 µm; Figure S5: Effect of different florfenicol concentrations ranging from 0.25 µg/mL to 2 µg/ml on the growth of *Aeromonas salmonicida* JF2267; Table S1: Effect of iron-limitation, elevated incubation temperature and florfenicol antibiotics on the subcellular proteomes and the vesiculation of the fish pathogen *Aeromonas salmonicida*; Table S2: Effect of iron-limitation, elevated incubation temperature and florfenicol antibiotics on the subcellular proteomes and the vesiculation of the fish pathogen *Aeromonas salmonicida*; Table S3: Effect of iron-limitation, elevated incubation temperature and florfenicol antibiotics on the subcellular proteomes and the vesiculation of the fish pathogen *Aeromonas salmonicida*.
