# Supplementary material for: Effect of Iron Limitation, Elevated Temperature, and Florfenicol on the Proteome and Vesiculation of the Fish Pathogen Aeromonas salmonicida
Source: Microorganisms. 2022 Aug 27;10(9):1735. doi: 10.3390/microorganisms10091735 (PMC9503180; doi:10.3390/microorganisms10091735)
Supplement: Supplementary file 1 [file microorganisms-10-01735-s001.zip › Supplement_figures.pdf]

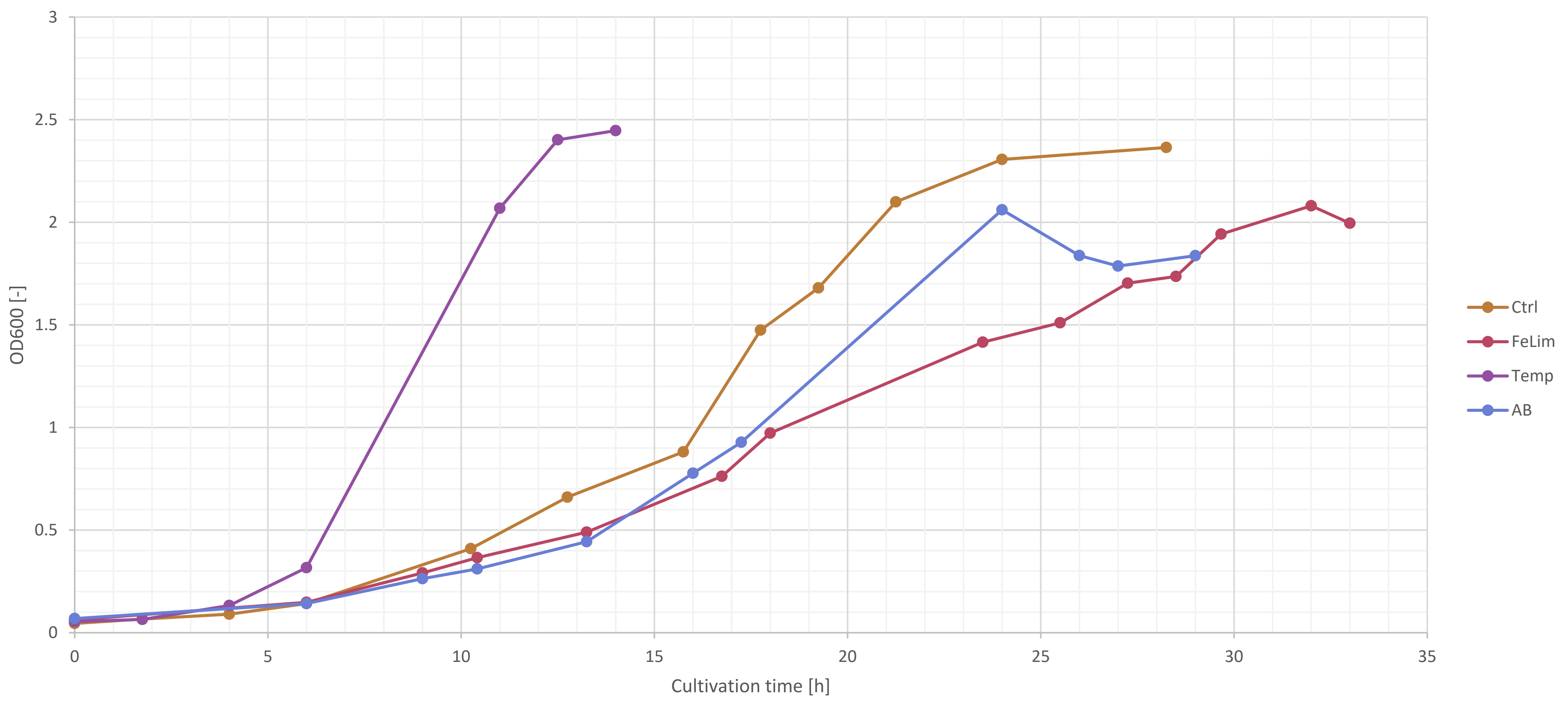

Supplementary Figure S1: Growth curves of *Aeromonas salmonicida* grown under different stress conditions.

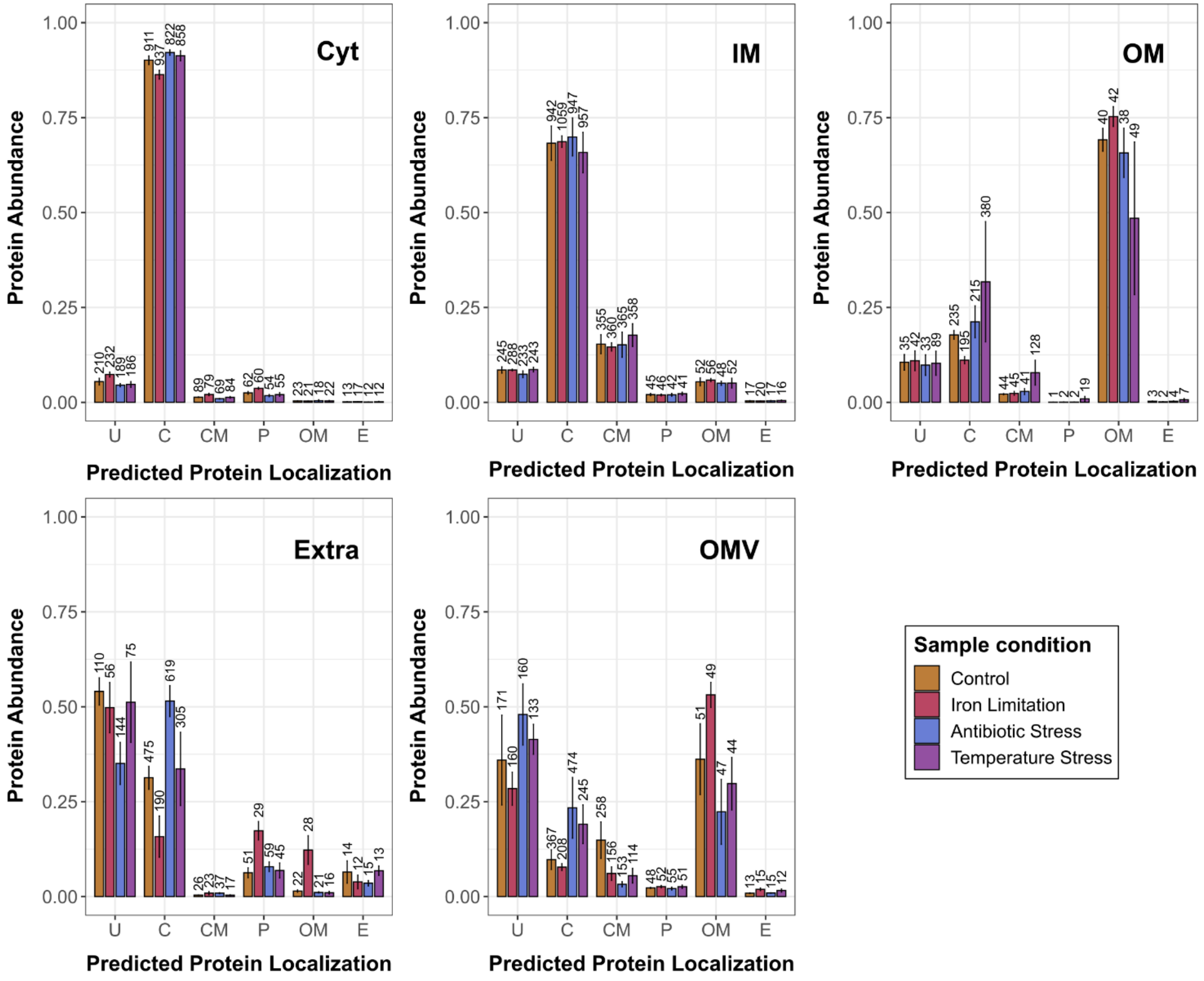

Supplementary Figure S2: Proteins quantified in the applied conditions were clustered according to their PSORTb predicted protein localization (U – unknown; C – cytoplasmic; CM – cytoplasmic membrane; P – periplasmic; OM – outer membrane; E – extracellular) for each of the enriched subcellular fractions. The protein abundancies of these clusters were normalized and the distribution within the enriched subcellular fractions was plotted. The number of quantified proteins in the experimental condition is indicated above the bars. Error bars indicate standard deviations between replicates (n = 4).

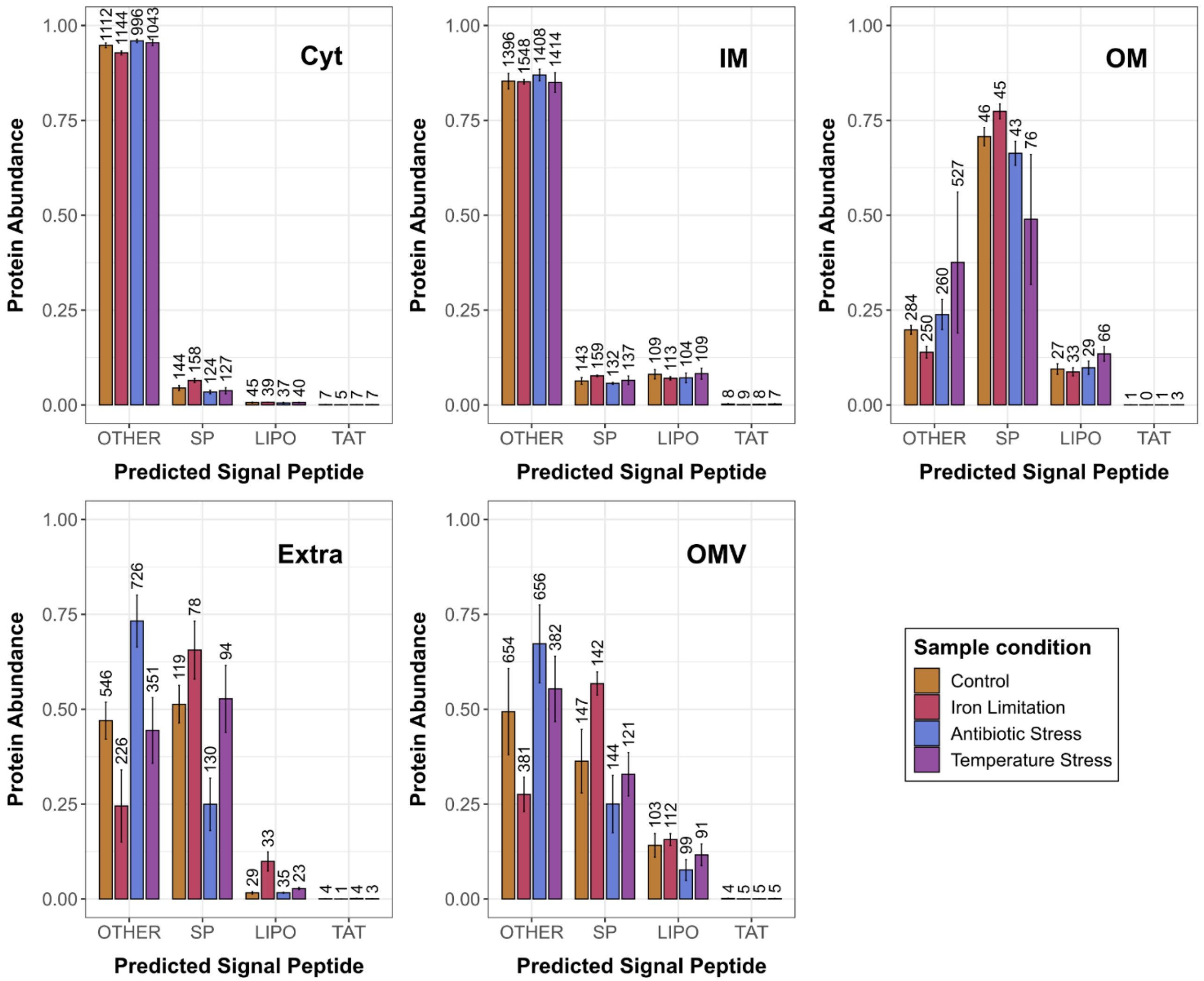

Supplementary Figure S3: Quantified proteins were clustered according to their SignalP predicted presence of signal pep-tides (OTHER – no predicted signal peptide; SP - Sec/SPI: "standard" secretory signal peptides transported by the Sec translocon and cleaved by signal peptidase I; LIPO - Sec/SPII: lipoprotein signal peptides transported by the Sec translocon and cleaved by signal peptidase II; TAT - Tat/SPI: Tat signal peptides transported by the Tat translocon and cleaved by signal peptidase). The protein abundancies of these clusters were normalized and the distribution within the enriched subcellular fractions of the applied conditions were plotted. of the applied conditions within the enriched subcellular fractions (A – Cytosol; B – Inner membrane; C – Outer membrane; D – Extracellular; E – Outer membrane vesicle). The number of quantified proteins within the enriched fractions are indicated above the bars. Error bars indicate standard deviations between replicates.

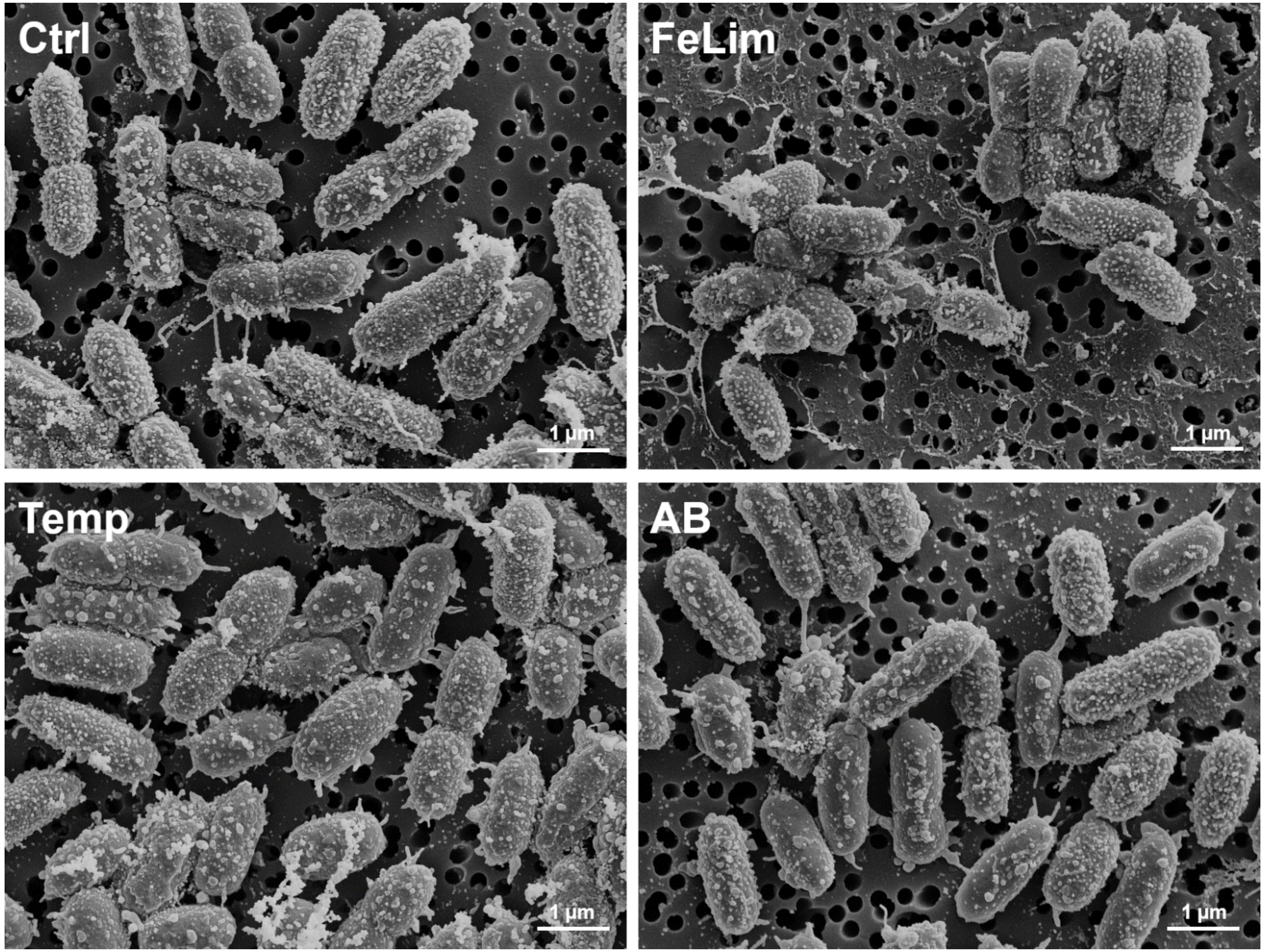

Supplementary Figure S4: Scanning electron micrographs of *Aeromonas salmonicida* under control (Ctrl), iron limitation (FeLim), elevated temperature (Temp) and antibiotic stress (AB) conditions. Scale bars = 1 µm.

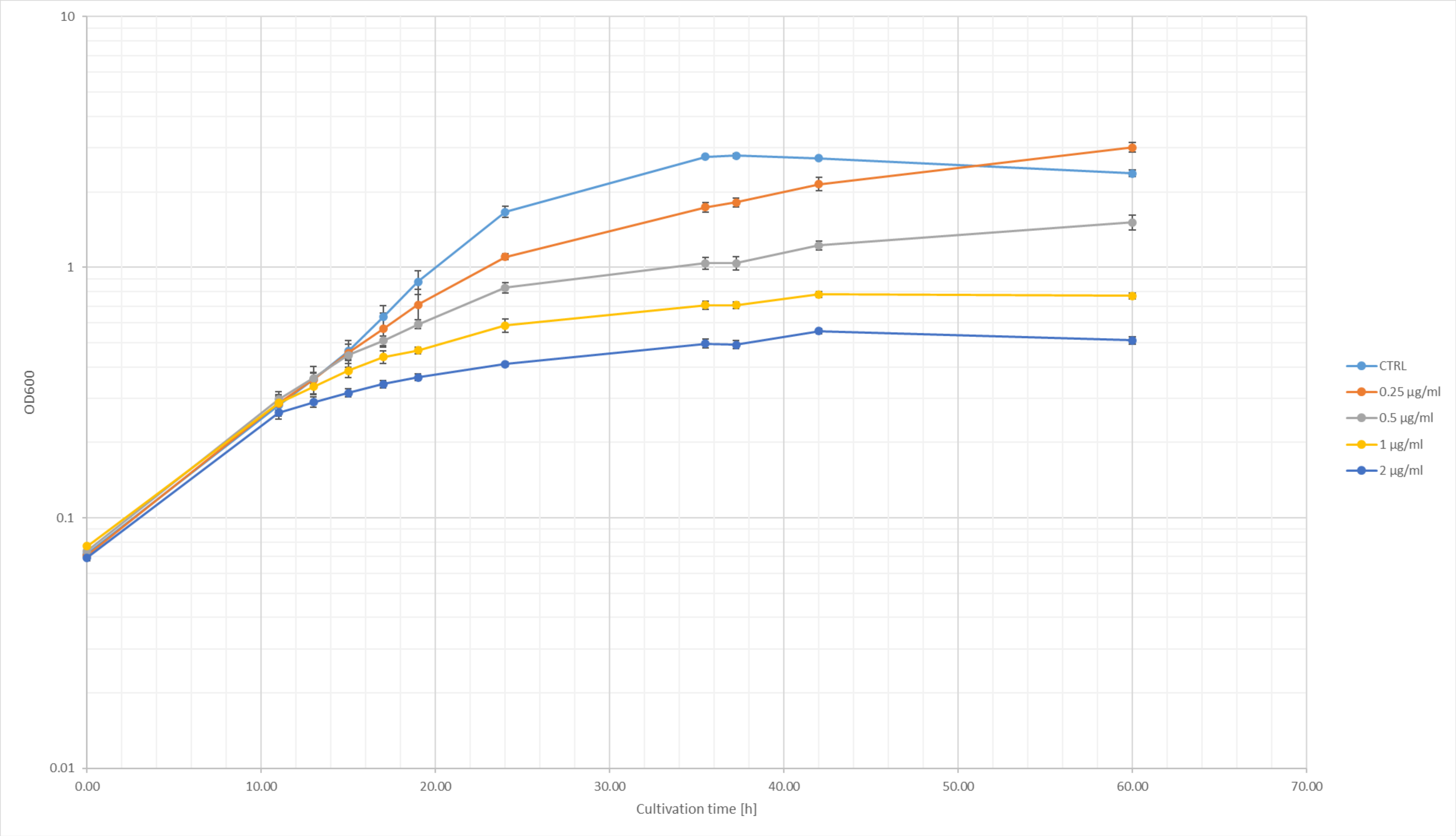

Supplementary Figure S5: Effect of different florfenicol concentrations ranging from 0.25 µg/ml to 2 µg/ml on the growth of *Aeromonas salmonicida* JF2267.
